# Modulation of duodenal and jejunal microbiota by rifaximin in mice with CCl_4_-induced liver fibrosis

**DOI:** 10.1186/s13099-023-00541-4

**Published:** 2023-03-21

**Authors:** Kazuhiko Ikeuchi, Takeya Tsutsumi, Aya Ishizaka, Taketoshi Mizutani, Ayako Sedohara, Michiko Koga, Satoru Tamaoki, Hiroshi Yotsuyanagi

**Affiliations:** 1 Division of Infectious Diseases, Advanced Clinical Research Center, Institute of Medical Science, The University of Tokyo, 4-6-1 Shirokanedai, Minato-Ku, Tokyo, 108-8639 Japan; 2 Department of Infection Control and Prevention, The University of Tokyo, 7-3-1 Hongo, Bunkyo-Ku, Tokyo, 113-8655 Japan; 3 Medical Affairs Department, ASKA Pharmaceutical Co., Ltd., 2-5-1, Shibaura, Minato-Ku, Tokyo, 108-8532 Japan

**Keywords:** Microbiome, Liver fibrosis, Small intestine, Hepatic encephalopathy, Lactobacillaceae, Firmicutes, Bacteroidetes

## Abstract

**Background:**

Rifaximin is a poorly absorbed broad-spectrum antibiotic used for hepatic encephalopathy. Although increased Lactobacillaceae and decreased Bacteroidetes abundance are characteristic of hepatic encephalopathy, rifaximin does not dramatically alter the stool microbiota. As the antimicrobial effect of rifaximin increases by micellization with bile acids, we hypothesized that rifaximin alters the microbiota in the duodenum and jejunum, where the levels of bile acids are abundant.

**Methods and results:**

Eight-week-old BALB/c mice were injected with carbon tetrachloride (CCl_4_) intraperitoneally for 12 weeks to induce liver fibrosis. The mice were grouped into the control (n = 9), CCl_4_ (n = 13), and rifaximin group in which mice were treated with rifaximin for two weeks after CCl_4_ administration (n = 13). We analyzed the microbiota of the duodenum, jejunum, ileum, cecum, and stool using 16S ribosomal RNA gene analysis. The content of Lactobacillaceae, the most abundant bacterial family in the duodenum and small intestine, increased in the CCl_4_ group, especially in the jejunum (median 67.0% vs 87.8%, p = 0.03). Rifaximin significantly decreased Lactobacillaceae content in the duodenum (median 79.4% vs 19.0%, p = 0.006) and jejunum (median 87.8% vs 61.3%, p = 0.03), but not in the ileum, cecum, and stool. Bacteroidetes abundance tended to decrease on CCl_4_ administration and increased following rifaximin treatment in the duodenum and jejunum. S24_7, the most abundant family in Bacteroidetes, demonstrated a significant inverse correlation with Lactobacillaceae (duodenum, r = − 0.61, p < 0.001; jejunum, r = − 0.72, p < 0.001). In the ileum, cecum, and stool, the effect of rifaximin on the microbiota was minimal, with changes within the same phylum. The percentage of bacterial families, such as Lactobacillaceae and S24_7 in the duodenum and small intestine, did not correlate with that in the stool.

**Conclusions:**

The abundance of Lactobacillaceae increased in the jejunum of mice with CCl_4_-induced liver fibrosis, while rifaximin significantly reduced it in the duodenum and jejunum. Thus, rifaximin possibly exerts its effect by altering the duodenal and jejunal microbiota. Furthermore, changes in the duodenal and small intestinal microbiota were not associated with that of stool, suggesting that the analysis of stool microbiota is insufficient to evaluate upper intestinal microbiota.

**Supplementary Information:**

The online version contains supplementary material available at 10.1186/s13099-023-00541-4.

## Background

Rifaximin is a semisynthetic antibiotic derived by modifying rifamycin to achieve low intestinal absorption (< 0.4%) [1]. Rifaximin inhibits RNA synthesis by binding the β subunit of DNA-dependent RNA polymerase and has broad-spectrum activity against aerobic and anaerobic bacteria [1, 2]. Although enteric microorganisms rapidly develop antibiotic resistance, rifaximin can achieve high concentration in the gastrointestinal tract and remain effective for a long time [2]. Furthermore, its low absorption rate results in low toxicity and minimal side effects [2].

In the United States, rifaximin has been approved for the treatment of hepatic encephalopathy, irritable bowel syndrome (IBS), and travelers’ diarrhea, all of which are associated with small intestinal microbiota [3]. Hepatic encephalopathy, a complication of end-stage liver cirrhosis, is a reversible neurological disorder caused by the ammonia produced from urea and amino acids by the host's intestinal tissues and microorganisms [4]. In patients with cirrhosis, decreased intestinal motility and reduced levels of bile acids and gastric acids lead to dysbiosis and small intestinal bacterial overgrowth (SIBO), inducing hyperammonemia. Dysbiosis also leads to a leaky gut and increases endotoxin levels in the portal vein, inducing hepatic inflammation via toll-like receptors 4 and 9 [5, 6]. In the stool of patients with hepatic encephalopathy, the abundance of Lactobacillaceae, Streptococcacae, and Enterobacteriaceae are increased, while that of Bacteroidaceae, Lachospiraceae, and Ruminococcaeae decreased [6–9]. Lactobacillaceae is a beneficial bacterial family, but is known to inversely increase in many hepatic diseases and is closely related to the development of hepatic encephalopathy [10–12]. Rifaximin has been reported to improve the symptoms of hepatic encephalopathy [13, 14], hyperammonemia [13, 14], endotoxemia [15], hyper-inflammation [16], and SIBO [17]. It is suggested that rifaximin exerts these beneficial effects by improving dysbiosis.

Interestingly, rifaximin does not significantly alter the stool microbiota, although it is a non-absorbable antimicrobial agent. In a study of 20 patients with liver cirrhosis treated with rifaximin for four weeks, symptoms of hepatic encephalopathy and serum ammonia levels improved, but there was no significant change in the fecal microbiota [18]. Similar results have been reported in animal models [19]. Therefore, the mechanism of action of rifaximin is not clear, and it is speculated that rifaximin exerts its action by affecting bacterial functions such as toxin production and mucosal adhesion [20].

Further, it has been hypothesized that the efficacy of rifaximin varies depending on the site of the intestinal tract. Although rifaximin is water-insoluble, its solubility is significantly improved by micellization with bile acids [21]. In vitro, it has been reported that the antimicrobial activity of rifaximin increases in the presence of bile acids [21]. Bile acids are discharged into the intestinal lumen in the duodenum, and most are actively absorbed in the ileum, with only about 5% being excreted in the stool [22]. Therefore, we hypothesized that rifaximin alters the duodenal and small intestinal microbiota associated with diseases. This hypothesis is supported by the fact that hepatic encephalopathy and IBS are accompanied by SIBO [23], and that enterotoxigenic *Escherichia coli* (ETEC), the most frequent causative bacterium of traveler's diarrhea, also adheres to the small intestine [20].

As it is difficult to evaluate the small intestinal specimen in humans, we used a mouse model of carbon tetrachloride (CCl_4_)-induced liver fibrosis. CCl_4_ is metabolized in the liver to trichloromethyl radical and trichloromethyl peroxy radicals, inducing liver damage [24]. CCl_4_-induced liver fibrosis is a common animal model of liver fibrosis and causes dysbiosis, such as increased Lactobacillaceae and decreased Bacteroidetes content, similar to that in human hepatic diseases [25, 26].

In this study, we aimed to investigate the effect of rifaximin on the dysbiosis caused by liver fibrosis in each region of the gastrointestinal tract. Using mice with CCl_4_-induced liver fibrosis, we evaluated the microbiota of the duodenum, jejunum, ileum, cecum, and stool by 16S ribosomal RNA (16S rRNA) gene analysis.

## Results

### *CCl*_*4*_*-induced liver fibrosis and increased gut permeability*

**Fig. 1:**
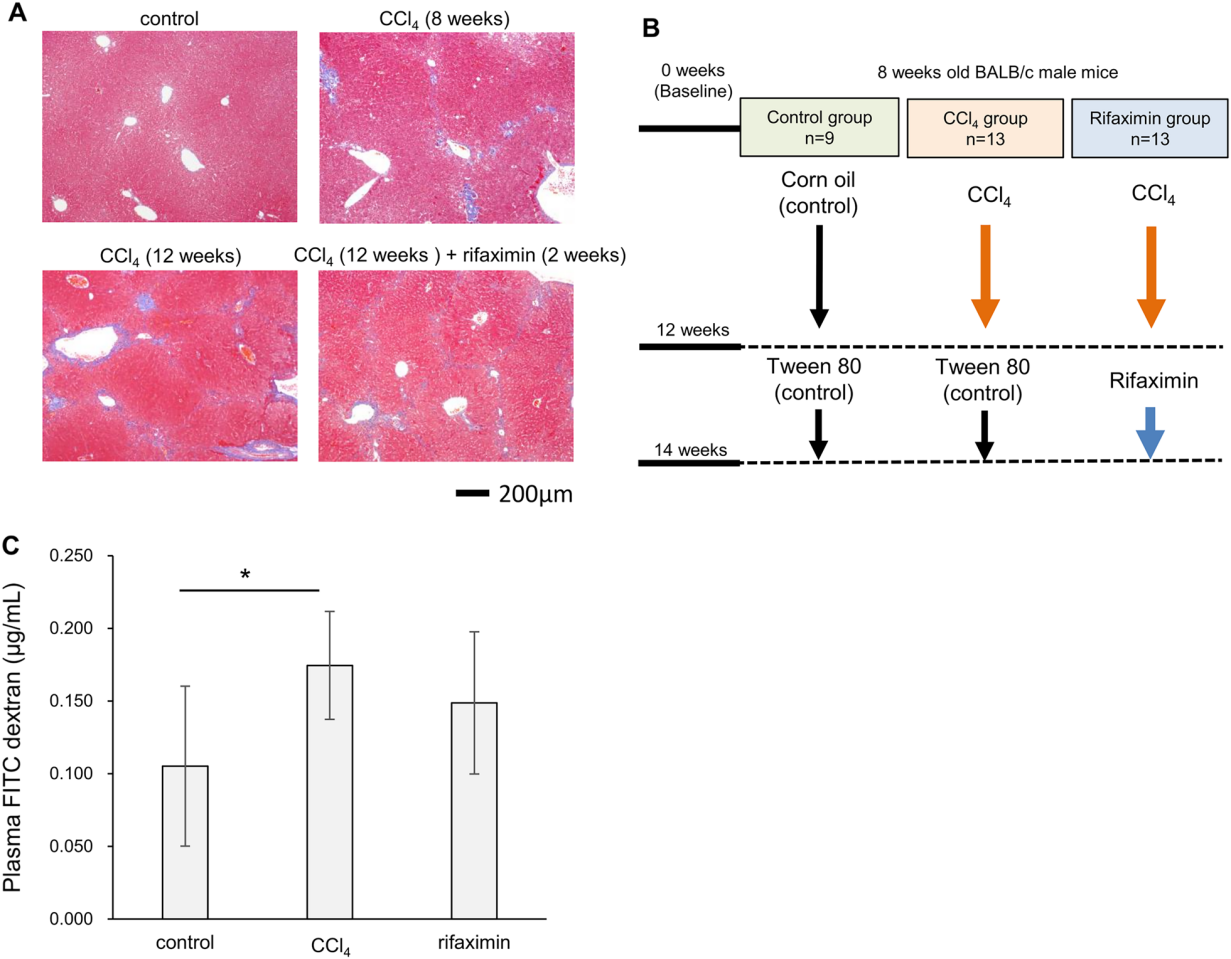
Study design. **A** Pathological images of the liver with Masson trichrome staining. Control mice showed no fibrosis. After 8 weeks of CCl_4_ administration, slight bridging fibrosis was observed between the portal area (preliminary experiment). After 12 weeks of CCl_4_ administration, bridging fibrosis was more significant and showed the Desmet-Scheuer scoring Stages 2–3. Rifaximin treatment for 2 weeks did not improve the fibrosis. **B** Mice in the control group were treated with corn oil and Tween-80. Mice in the CCl_4_ group were treated with CCl_4_ and Tween-80. Mice in the rifaximin group were treated with CCl_4_ and rifaximin. **C** FITC-dextran gut permeability test. Average and 95% confidence interval are shown. CCl_4_, carbon tetrachloride. *p < 0.05, compared with the CCl_4_ group

We administered CCl_4_ or corn oil to 8-week-old BALB/c mice. Figure 1A shows pathological images of the liver with Masson trichrome staining. Compared to the 8-week administration of CCl_4_ in the preliminary experiment, the 12-week administration showed more severe fibrosis in the Desmet-Scheuer scoring Stages 2–3 [27].

Based on these results, we analyzed three groups (Fig. 1B): (1) a control group in which mice were treated with corn oil for 12 weeks followed by 2 weeks of Tween-80 (n = 9), (2) a CCl_4_ group in which mice were treated with CCl_4_ for 12 weeks followed by 2 weeks of Tween-80 (n = 13), and (3) a rifaximin group in which mice were treated with CCl_4_ for 12 weeks followed by 2 weeks of rifaximin (n = 13). Two weeks of rifaximin administration did not alter the pathological liver fibrosis (Fig. 1A).

To examine the effect of CCl_4_ and rifaximin on intestinal permeability, the FITC dextran intestinal permeability test was performed. The dextran concentration, estimated from the fluorescence of FITC in plasma, is shown in Fig. 1C. Plasma dextran concentrations were significantly higher in the CCl_4_ group compared to those in the control (control vs CCl_4_, average 0.11 vs 0.17 µg/mL, p = 0.03), while the rifaximin group did not show a significant decrease (average 0.15 µg/mL, p = 0.36).

### Sufficient reads were derived even in the duodenum and small intestine

We then performed next-generation sequencing of the V3 and V4 regions of the 16S rRNA gene for the intestinal components and stool. To evaluate the number of reads, we checked the alpha rarefaction curve (Additional file 1: Figure S1). The median reads were 20,301 (interquartile rage [IQR], 14,849–26,607). The duodenum and small intestine had relatively low numbers of reads assigned to bacteria due to contamination with host DNA because of their low bacterial abundance, but the minimum number of reads was 1387, which was considered sufficient for the analysis of bacterial flora.

### Rifaximin-induced changes in alpha and beta diversity

First, we analyzed the alpha and beta diversity of samples with > 3000 reads where the operational taxonomic units (OTUs) had not reached a plateau from the diversity analyses (duodenum, n = 1; jejunum, n = 4; ileum, n = 1). This exclusion of the data did not significantly affect the below results.

**Fig. 2:**
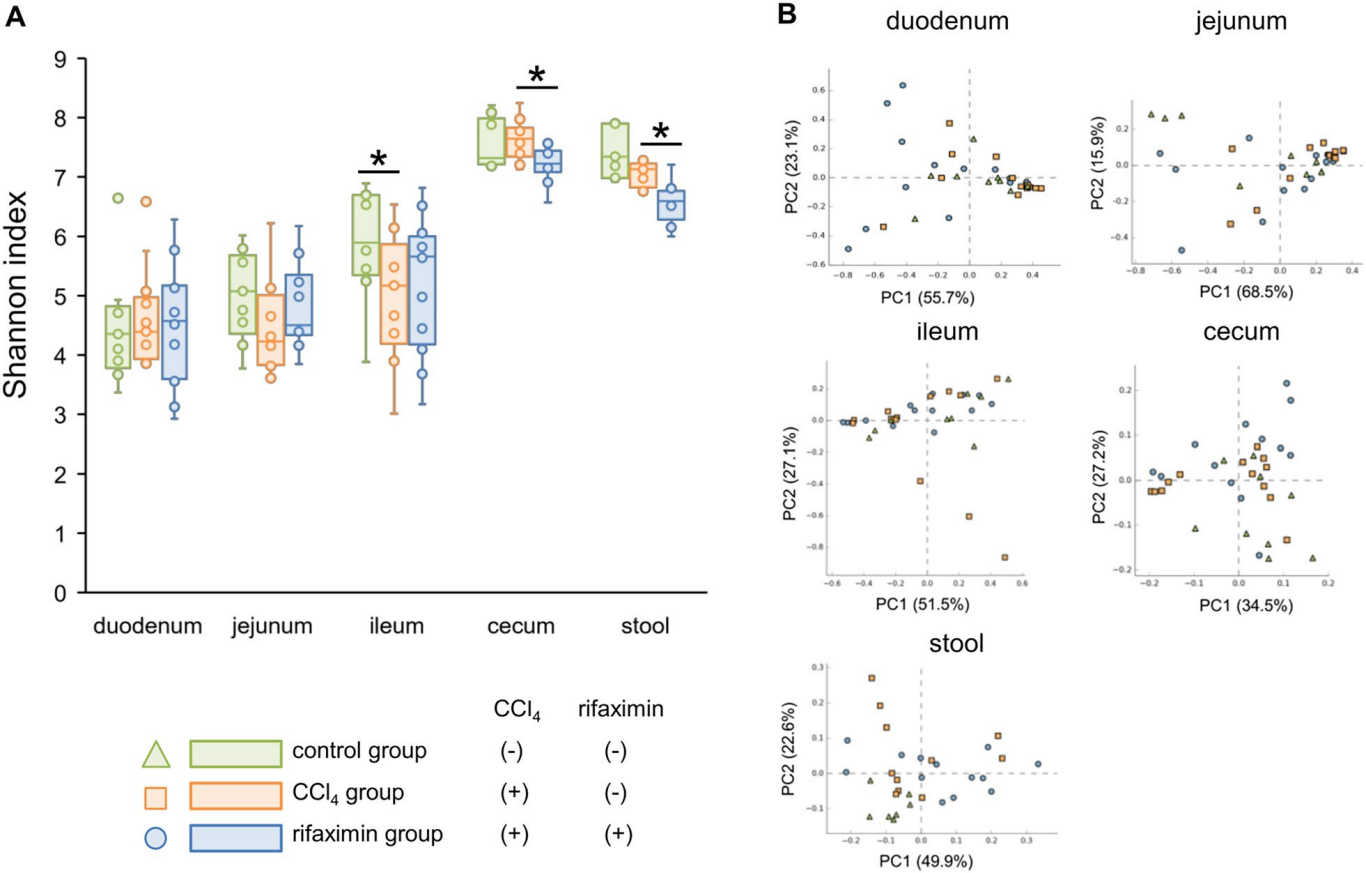
Alpha and beta diversity of each intestinal site. **A** Shannon index of each intestinal site. **B** Principal coordinate analysis of each intestinal site. Green triangles are the control group. Red squares are the CCl_4_ group. Blue circles are the rifaximin group. CCl_4_, carbon tetrachloride; PC, Principal Coordinate. *p < 0.05, compared with the CCl_4_ group

The Shannon index is shown in Fig. 2A. In the jejunum, the Shannon index tended to decrease in the CCl_4_ group (control vs CCl_4_, median [IQR], 5.1 [4.6–5.6] vs 4.3 [3.9–5.1], p = 0.10), and tended to increase in the rifaximin group (4.5 [4.4–5.2], p = 0.16). In the ileum, the Shannon index was significantly higher in the control group than in the CCl_4_ group (control vs CCl_4_, 5.9 [5.5–6.7] vs 5.2 [4.4–5.6], p = 0.04). In the cecum and stool, the Shannon index was significantly decreased by rifaximin administration (CCl_4_ vs rifaximin, cecum, 7.6 [7.4–7.8] vs 7.2 [7.1–7.4], p = 0.003; stool, 7.1 [6.9–7.2] vs 6.6 [6.4–6.7], p < 0.001).

A principal coordinate analysis plot is shown in Fig. 2B. Weighted Unifrac analysis showed that the rifaximin and CCl_4_ groups had different flora structures in the duodenum (p = 0.04), jejunum (p = 0.006), cecum (p = 0.02), and stool (p = 0.003), but not in the ileum (p = 0.78). Comparing the control and CCl_4_ groups, only the stool showed difference (p = 0.002).

### The duodenum and small intestine had fewer bacterial families than the cecum and stool

**Fig. 3:**
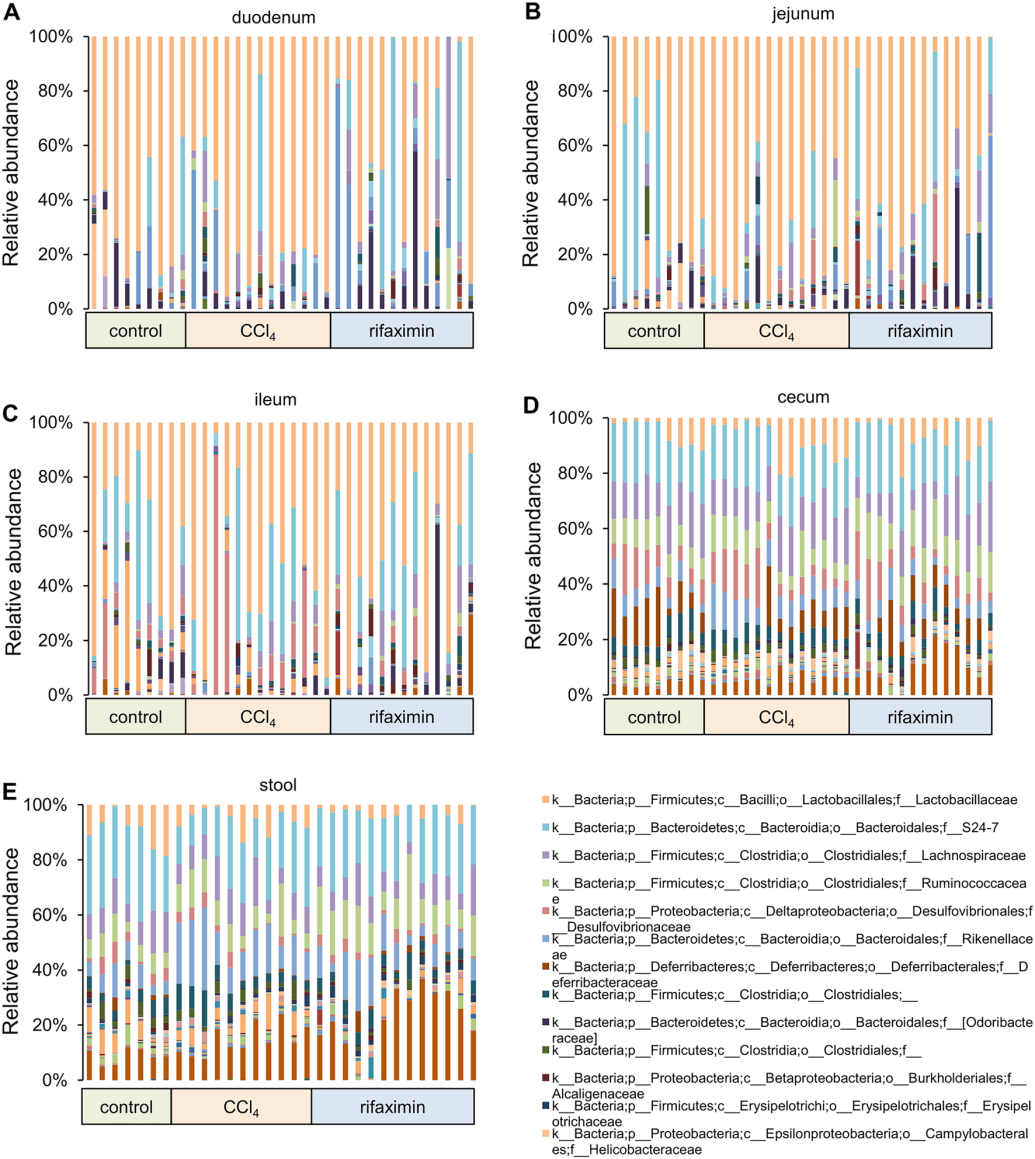
Relative abundance of bacterial families in each sample. CCl_4_, carbon tetrachloride; c, class; f, family; o, order; p, phylum

The relative abundance of bacterial families in each sample is shown in Fig. 3. Compared to the cecum and stool, the duodenum and small intestine contained fewer bacterial families and had much greater variation among samples even in the same group. Bacterial families with a median relative abundance greater than 1% in at least the duodenum, jejunum, or ileum were extracted; the results showed that only six bacterial families accounted for a median of 95.9% (IQR 88.1–98.5%) of the duodenal microbiota, 94.9% (IQR 89.3–97.6%) of the jejunal microbiota, and 86.9% (IQR 77.8–94.2%) of the ileal microbiota: S24_7, Lactobacillaceae, Lachnospiraceae, Streptococcaceae, Enterobacteriaceae, and Desulfovibrionaceae.

On the contrary, more bacterial families were observed in the cecum and stool. S24_7, Bacteroidaceae, Rikenellaceae, Paraprevotellaceae, Deferribacteraceae, Lactobacillaceae, Lachnospiraceae, Ruminococcaceae, unassigned Clostridiales, Erysipelotrichaceae, Desulfovibrionaceae, Helicobacteraceae, and F16 had a relative median abundance greater than 1% in the cecum or stool. These bacterial families accounted for a median of 94.4% (IQR, 93.4–96.0%) of the cecal microbiota, and a median of 94.4% (IQR, 92.2–95.8%) of the stool microbiota.

### Rifaximin significantly decreased Lactobacillaceae abundance in the duodenum and jejunum

**Fig. 4:**
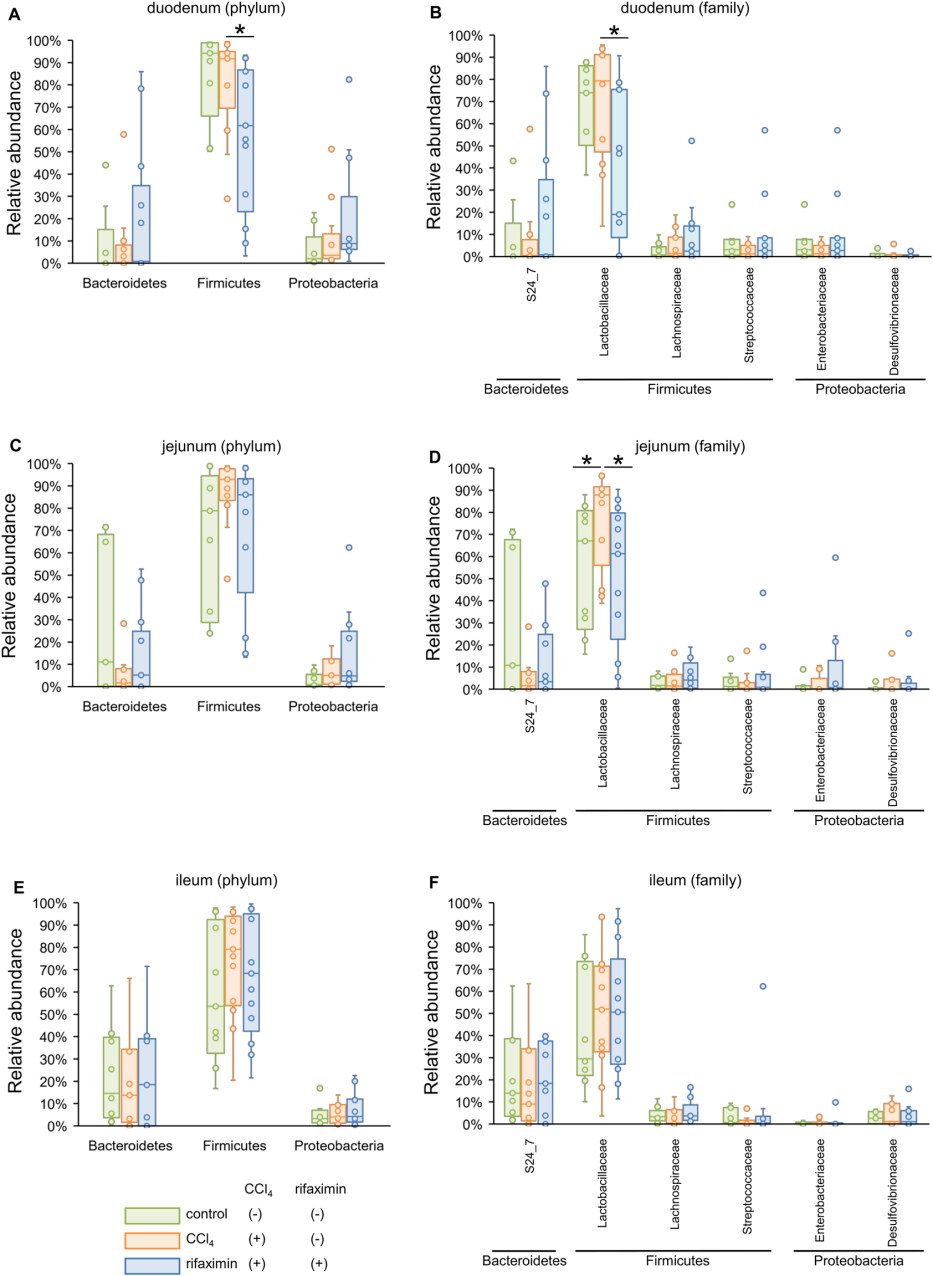

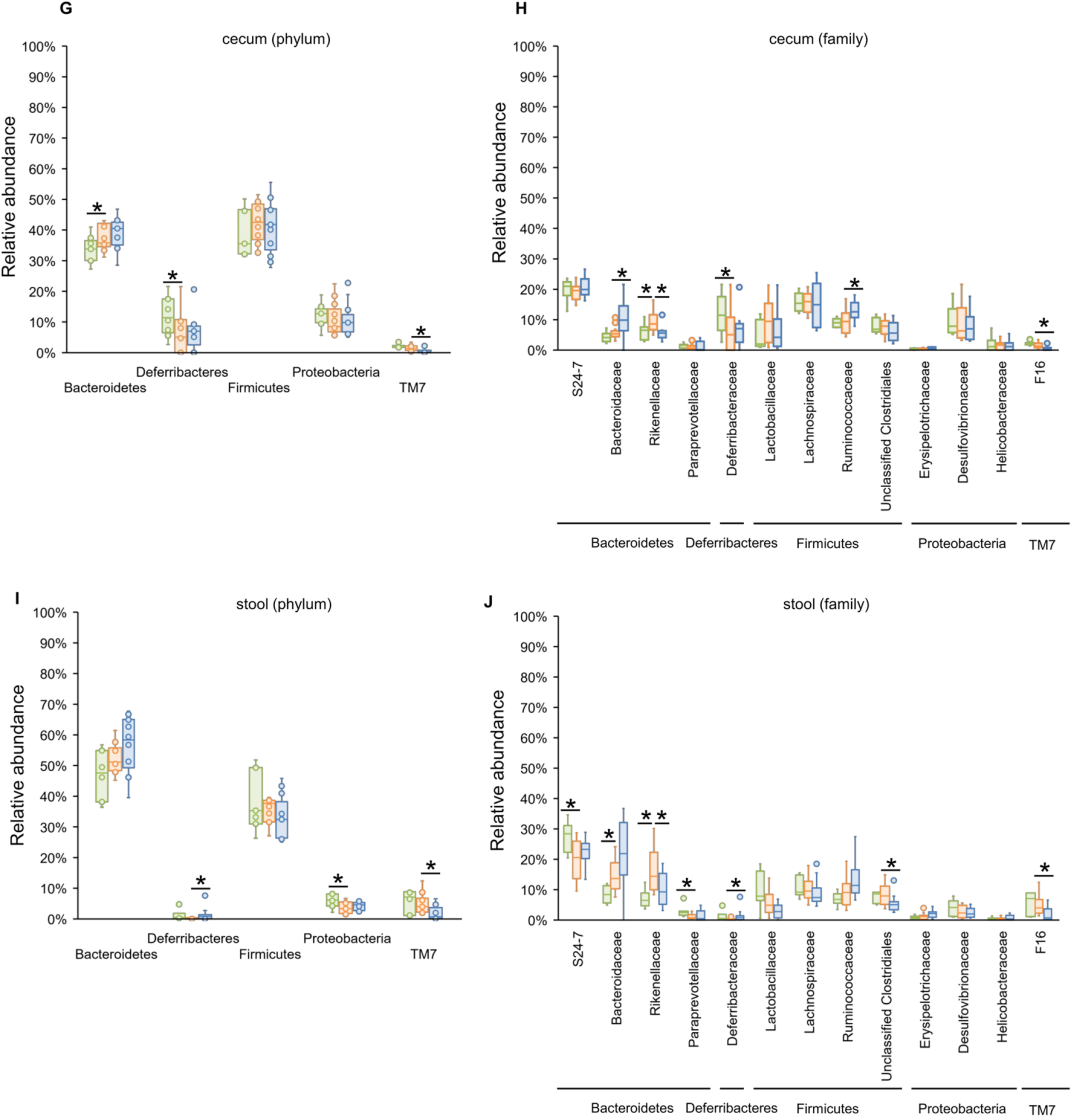
The box plot of bacterial phylum and family with a high relative abundance. The bacterial phyla and families with a median relative abundance of > 1% in at least the duodenum, jejunum, or ileum are shown in **A** to **F**. The bacterial families with a median relative abundance of > 1% in at least the cecum or stool, are shown in G to J. **A** microbiota of the duodenum at the phylum level. **B** microbiota of the duodenum at the family level. **C** microbiota of the jejunum at the phylum level. **D** microbiota of the jejunum at the family level. **E** microbiota of the ileum at the phylum level. **F** microbiota of the ileum at the family level. **G** microbiota of the cecum at the phylum level. **H** microbiota of the cecum at the family level. I microbiota of the stool at the phylum level. J: microbiota of the stool at the family level. * p < 0.05, and LDA > 3.5, compared with the CCl_4_ group. CCl_4_, carbon tetrachloride; LDA, linear discriminant analysis

Owing to the large variation in relative abundance among samples in the duodenum and small intestine, we illustrated the box plot (median [IQR]) of bacterial relative abundance at the phylum and family levels, as shown in Fig. 4. All bacteria with statistical significance in the linear discriminant analysis effect size (LEfSe) analysis are shown in Additional file 2: Table S1–S10.

In the duodenum (Fig. 4A and B), although there was no statistically significant change after CCl_4_ administration, an abundance of Firmicutes, especially Lactobacillaceae, significantly decreased in the rifaximin group (CCl_4_ vs rifaximin, 79.4% [52.9–90.8%] vs 19.0% [15.4–75.4%], p = 0.006).

In the jejunum (Fig. 4C and D), the abundance of Lactobacillaceae significantly increased in the CCl_4_ group (control vs CCl_4_, 67.0% [32.2–78.6%] vs 87.8% [67.5–90.6%], p = 0.03) and significantly decreased in the rifaximin group (61.3% [33.7–77.4%], p = 0.03); similar results were observed in the duodenum. In contrast, S24_7 demonstrated a decreasing trend in the CCl_4_ group (control vs CCl_4_, 10.8% [0.2–64.2%] vs 1.7% [0.2–7.4%], p = 0.18) and increasing trend in the rifaximin group (3.4% [0.1–20.6%], p = 0.50).

In the ileum (Fig. 4E and F), Lactobacillaceae abundance tended to increase in the CCl_4_ group (control vs CCl_4_, 29.5% [24.5–71.0%] vs 52.0% [34.3–70.2%], p = 0.43) while the rifaximin group showed little change (50.6% [29.3–64.5%], p = 0.69). Although the relative abundance was low, Clostridiaceae (CCl_4_ vs rifaximin, 0.03% [0–3.5%] vs 0% [0–0%], p = 0.008) and F16 (CCl_4_ vs rifaximin, 1.4% [0.1–4.3%] vs 0% [0–0.8%], p = 0.05) were significantly less in the rifaximin group compared to the CCl_4_ group (Additional file 1: Table S6).

### The effect of rifaximin on the cecal and stool microbiota was minimal

In the cecum (Fig. 4G and H), there were only changes within the same phylum (e.g., Bacteroidaceae and Rikenellaceae in the phylum Bacteroidetes) or in bacteria with low relative abundance. Ruminococcaceae abundance was significantly increased in the rifaximin group (CCl_4_ vs rifaximin, 9.4% [5.8–11.8%] vs 12.6% [11.0–14.5%], p = 0.03), and F16 was significantly decreased in the rifaximin group as in the ileum (CCl_4_ vs rifaximin, 1.3% [0.8–2.2%] vs 0.3% [0.02–0.9%], p = 0.009).

In the stool (Fig. 4I and J), the CCl_4_ group showed some changes in bacterial proportions; however, the changes were only within the same phylum (e.g., S24-7, Paraprevotellaceae, Rikenellaceae, and Bacteroidaceae) and bacteria with low relative abundance (Additional file 1: Table S9). Rifaximin did not alter the microbiota remarkably but an increased abundance of the genus *Oscillospira* in Ruminococcaceae (CCl_4_ vs rifaximin, 5.1% [3.1–7.4%] vs 10.6% [8.2–12.1%], p = 0.004) and decreased F16 (CCl_4_ vs rifaximin, 4.0% [2.5–6.4%] vs 0.7% [0.2–2.6%], p = 0.007) as in the cecum.

### The relative abundance of bacterial families in the duodenum and small intestines poorly correlated with that in the stool

**Fig. 5:**
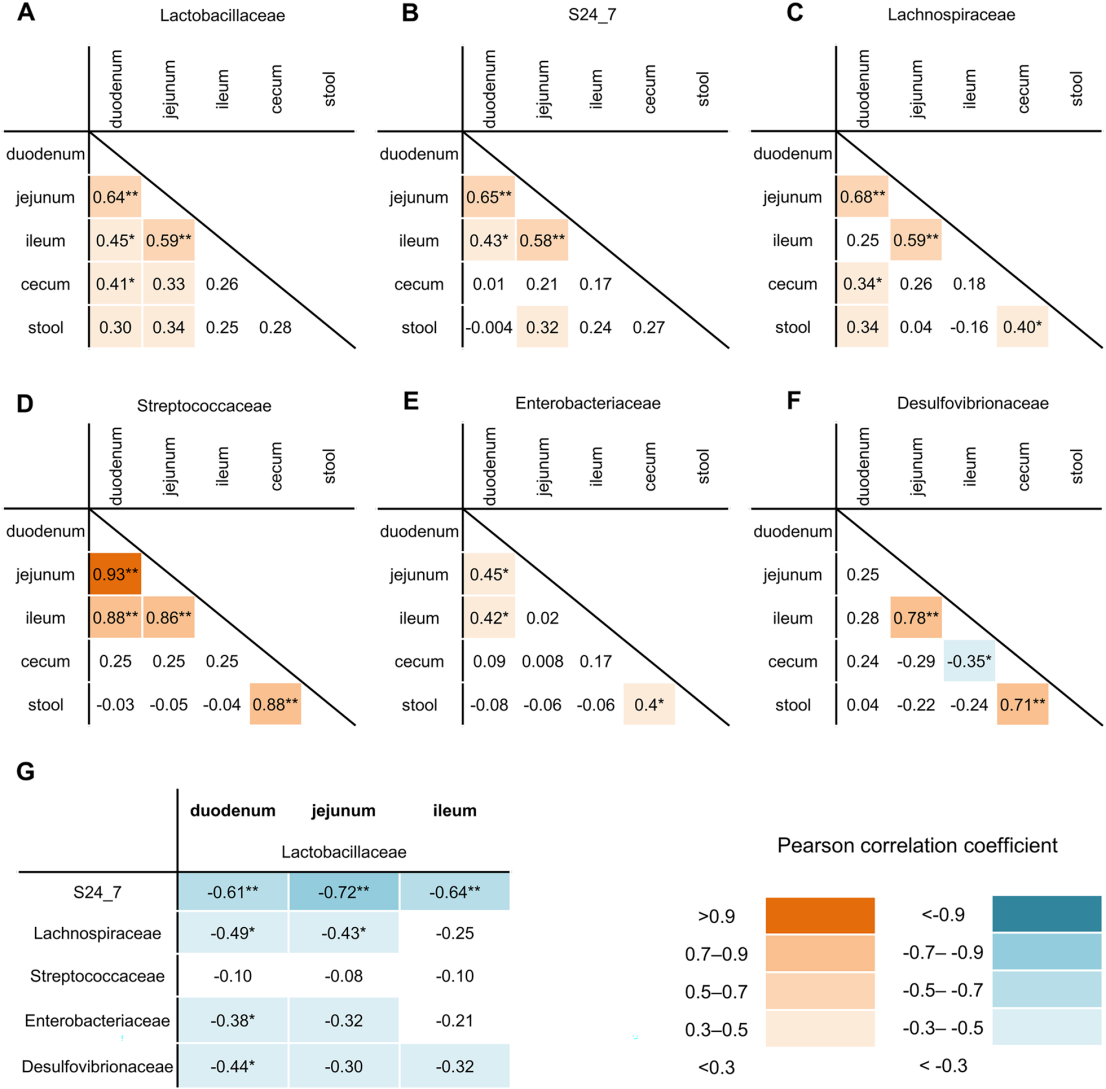
Correlation between bacterial families. Pearson’s correlation coefficient between sites of the intestinal tract is shown in each bacterial family. **A** Lactobacillaceae, **B** S24_7, **C** Lachnospiraceae, **D** Streptococcaceae, **E** Enterobacteriaceae, **F** Desulfovibrionaceae. **G** The correlation between Lactobacillaceae and other bacterial families in the duodenum, jejunum, and ileum. *p < 0.05, **p < 0.001

To assess whether the microbiota of the duodenum and small intestine can be predicted from the microbiota of the stool, we correlated the proportion of bacteria between sites of the intestinal tract (Fig. 5A–F).

The relative abundance of most bacterial families correlated between the duodenum and jejunum (Lactobacillaceae, r = 0.64, p < 0.001; S24_7, r = 0.65, p < 0.001; Lachnospiraceae, r = 0.68, p < 0.001; Streptococcaceae, r = 0.93, p < 0.001; Enterobacteriaceae, r = 0.45, p = 0.006), and between the jejunum and ileum (Lactobacillaceae, r = 0.59, p < 0.001; S24_7, r = 0.58, p < 0.001; Lachnospiraceae, r = 0.59, p < 0.001; Streptococcaceae, r = 0.86, p < 0.001; Desulfovibrionaceae, r = 0.78, p < 0.001). However, the relative abundance of duodenal and small intestinal bacterial families did not significantly correlate with that of the stool.

### S24_7 was inversely correlated with Lactobacillaceae in the duodenum, jejunum and ileum,

Correlation analysis was performed between Lactobacillaceae, the most significantly altered bacterial family, and other bacterial families (Fig. 5-G). S24_7 had the strongest inverse correlation with Lactobacillaceae in the duodenum (r = − 0.61, p < 0.001), jejunum (r = − 0.72, p < 0.001), and ileum (r = − 0.64, p < 0.001). Lachnospiraceae had a weak inverse correlation with Lactobacillaceae in the duodenum (r =− 0.49, p = 0.003) and jejnum (r = − 0.43, p = 0.01). Desulfovibrionaceae (r = − 0.44, p = 0.008), and Enterobacteriaceae (r = − 0.38, p = 0.03) had a weak inverse correlation with Lactobacillaceae only in the duodenum. Streptococcaceae did not have significant inverse correlation in any of the sites.

### *The amount of Lactobacillaceae increased in the CCl*_*4*_* group and decreased in the rifaximin group*

To evaluate the bacterial amount in the intestinal tract, quantitative polymerase chain reaction for the 16S rRNA gene was performed on 15 jejunal (control, n = 4; CCl_4_, n = 5; rifaximin, n = 6) and 20 cecal (control, n = 4; CCl_4_, n = 7; rifaximin, n = 9) samples.

**Fig. 6:**
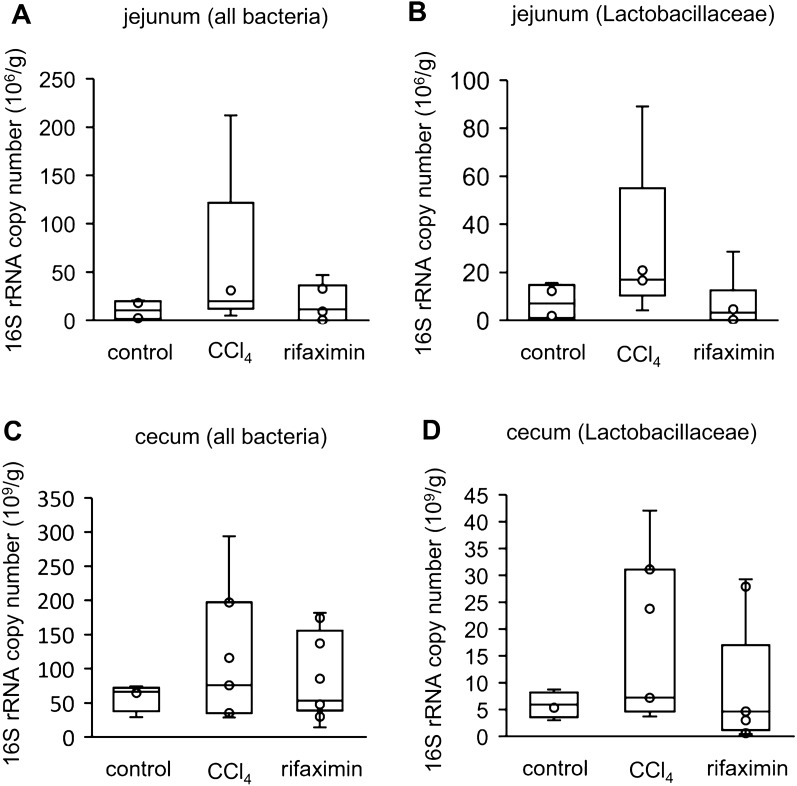
Quantitative 16S rRNA PCR. The number of 16S rRNA gene copies per intestinal contents (g) is shown. Fifteen jejunal (control, n = 4; CCl_4_, n = 5; rifaximin, n = 6) and 20 cecal (control, n = 4; CCl_4_, n = 7; rifaximin, n = 9) samples were analyzed. **A** total bacteria in the jejunum. **B** Lactobacillaceae in the jejunum. **C** total bacteria in the cecum. **D** Lactobacillaceae in the cecum. CCl_4_, carbon tetrachloride

Although the sample size was small, there was an overall increasing trend in the CCl_4_ group and a decreasing trend in the rifaximin group (Fig. 6). The most significant difference was in the amount of Lactobacillaceae in the jejunum (control vs CCl_4_, 7.0 [1.6–13.0] vs 16.9 [16.6–20.9] × 10^6^/g, p = 0.06; CCl_4_ vs rifaximin, 16.9 [16.6–20.9] vs 3.2 [0.6–6.5] × 10^6^/g, p = 0.13).

## Discussions

We showed that Lactobacillaceae, the most abundant bacterial family in the duodenum and small intestine, significantly increased in the jejunum with CCl_4_-induced liver fibrosis, and significantly decreased in the duodenum and jejunum with rifaximin treatment. S24_7, also known as Muribaculaceae, had a moderate inverse correlation with Lactobacillaceae in the duodenum, jejunum and ileum. Rifaximin altered the cecal and stool microbial composition less dramatically compared with the duodenum and jejunum. An abundance of major bacterial families in the small intestine, such as Lactobacillaceae, was decreased in the stool. Further, the percentage of bacterial families in the small intestine was not significantly correlated with that in the stool, indicating that the microbiota of the small intestine should be directly evaluated.

Rifaximin remarkably decreased Lactobacillaceae abundance in the duodenum and jejunum, and inversely increased that of Bacteroidetes. Although rifaximin has a broad-spectrum antibiotic effect against most intestinal bacteria, it has been reported that Gram-negative bacteria, such as Bacteroidetes, are more likely to acquire resistance to rifaximin than Gram-positive bacteria such as Firmicutes (e.g., Lactobacillaceae and Clostridiaceae) [28]. Our results are consistent with this previous report.

Our study also showed that Lactobacillaceae abundance markedly increased especially in the jejunum of CCl_4_-treated mice. In recent years, the increase of Lactobacillaceae in the stool was revealed to be associated with obesity [29, 30], diabetes [30], nonalcoholic fatty liver disease (NAFLD) [31], viral hepatitis [32], cirrhosis [30, 33], and hepatic encephalopathy [10–12]. Since Lactobacillaceae is a dominant bacterial family in the small intestine and is involved in the metabolism of glucose, amino acids, and bile acids, the small intestinal microbiota might be significant in these diseases [30, 34]. It has also been reported that Lactobacillaceae abundance in the cecum increased in mice with CCl_4_-induced liver fibrosis [25].

Lactobacillaceae content is known to increase especially in hepatic encephalopathy, which is the most common indication for rifaximin. An increasing Lactobacillaceae content at the onset of hepatic encephalopathy correlates with one-year mortality [11]. In an animal model of hepatic encephalopathy, Lactobacillaceae was correlated with brain inflammation [26]. Ammonia, the main cause of hepatic encephalopathy, is produced by the metabolism of amino acids in the small intestine by hosts and intestinal bacteria, such as Lactobacillaceae [4, 34]. Lactobacillaceae also has a specific urease active at low pH in the small intestine and can produce ammonia through uric acid degradation [35]. In addition, lactic acid produced by Lactobacillaceae exacerbates cerebral edema and contributes to the development of hepatic encephalopathy, along with ammonia [4, 36]. Although the mechanism of action of rifaximin in lowering blood ammonia and improving hepatic encephalopathy is still unclear, the decreased Lactobacillaceae abundance in the duodenum and jejunum might contribute to it.

With the decrease in Lactobacillaceae content by rifaximin administration, an abundance of S24_7, a family of Bacteroidetes increased in the duodenum and small intestine. The abundance of Bacteroidetes decreases in cirrhosis [7–9], and this decrease is a risk for hepatic encephalopathy [12]. S24_7 content decreases in both alcoholic and non-alcoholic hepatic fatty liver disease [37, 38]. Bacteroidetes produce short-chain fatty acids and exert anti-inflammatory effects [39], and the ratio of Bacteroidetes/Firmicutes in the stool, which is used as a marker of dysbiosis, decreased in hepatic diseases [8]. Although we could not evaluate the Bacteroidetes/Firmicutes ratio in the small intestine because the percentage of Bacteroidetes was 0% in many small intestinal samples, rifaximin-induced decreased Firmicutes and increased Bacteroidetes content may be beneficial against hepatic diseases.

In this study, several other bacteria were affected by rifaximin. Lachnospiraceae, which increased in the duodenum and jejunum with a decrease in Lactobacillaceae, and *Oscillospira* in Ruminococcaceae, which increased in the cecum and stool following rifaximin administration, are well-known bacteria that decrease in NAFLD, liver cirrhosis, hepatic encephalopathy, and CCl_4_-treated mice [7, 8, 26, 33, 40, 41]. Ruminococcaceae and Lachnospiraceae are typical beneficial bacteria that produce butyric acid [9], which may be a favorable change caused by rifaximin. F16 in the phylum TM7, which was decreased in the ileum, cecum, and stool by rifaximin, was a very small and difficult-to-cultivate bacterium. Although its function is not well-known, it has been reported to be associated with inflammatory bowel disease [42].

Although no previous reports have evaluated the effects of rifaximin on the microbiota from the duodenum to the stool, a few reports have examined its effects on the small intestine. In a study on stress-induced intestinal inflammation, rifaximin treatment increased Lactobacillaceae content in the terminal ileum of rats, but only the terminal ileum of three rats in the same cage was analyzed [43]. In another study on ethanol-induced liver injury mice, rifaximin decreased Firmicutes and increased Bacteroidetes abundance as in the present study [44]. Contrary to other hepatic diseases, Lactobacillaceae abundance is decreased in alcoholic hepatitis; in this study, its content was drastically reduced in the small intestine after ethanol administration, and the effect of rifaximin on Lactobacillaceae was unclear. Further study of the small intestinal microbiota may help predict the effects of rifaximin depending on the diseases.

Rifaximin is known to improve SIBO [17, 45]. In the present study, although the sample number was the small, bacterial amount, especially that of Lactobacillaceae in the jejunum, tended to increase in the CCl_4_ group and decrease in the rifaximin group. Rifaximin is known to reduce the number of bacteria in the duodenum [20], but its effect may depend on the bacterial species and requires further investigation.

Herein, rifaximin decreased the alpha diversity in the cecum and stool, a change that has been previously reported [46]. There was no statistically significant difference in alpha diversity in the duodenum and small intestine, likely because bacterial species were significantly fewer compared with the cecum and stool. Although it is unclear whether the reduction in stool alpha diversity has an unbeneficial effect, our results suggest that rifaximin may be more effective for diseases associated with the small intestinal microbiota than the colonic microbiota.

Two-week treatment of rifaximin did not improve pathological liver fibrosis in the present study. Previous reports showed that rifaximin improves systemic inflammation and endotoxemia [15, 16]. Hence, rifaximin may improve liver fibrosis mediated by the gut-liver axis on prolonged usage, and further studies are required.

This study had several limitations. First, the microbiota of mice is not the same as that of humans, and it is not clear whether the effect of rifaximin is the same in humans. However, the general trend of bacterial flora (e.g., Lactobacillaceae is dominant in the small intestine) is similar in humans and mice. Second, we did not analyze the correlation between the bacteria altered by rifaximin and other markers associated with hepatic diseases. A previous study showed that rifaximin improves CCl_4_-induced leaky gut [25]; however, the difference was not statistically significant in our study and we could not identify the causative bacteria. Although previous reports showed that rifaximin decreased the levels of inflammatory cytokines [16], the BALB/C mice used in this study were highly sensitive to CCl_4_ [24]due to which we used a low dose. Thus, there was no significant increase in TNF-α and IL-6 levels even in the CCl_4_ group (data not shown). However, previous reports have shown that rifaximin decreases levels of blood ammonia and inflammatory cytokines, and intestinal permeability, and the problem was that the mechanism could not be explained by changes in the stool microbiota.

## Conclusions

Rifaximin, a poorly absorbed antimicrobial agent, decreases Lactobacillaceae abundance, mainly in the duodenum and jejunum, where bile acids are abundant. This study elucidates the mechanism of action of rifaximin and highlights the importance of analyzing the microbiota of the small intestine directly.

## Methods

### Animals

BALB/c mice born in the Animal Center, Institute of Medical Science, University of Tokyo were used for the analysis. We only analyzed male mice, considering the influence of estrogen on the liver. All mice were group-housed (1–4 mice per cage) with a laboratory temperature of 22–24 °C and a 12 h light-dark cycle. Mice were fed a standard chow (CA-1) obtained from CLEA Japan (Tokyo, Japan).

### Materials

CCl_4_, corn oil, and Tween 80 were obtained from FUJIFILM Wako Chemicals (Osaka, Japan). Rifaximin was provided by ASKA Pharmaceutical Co., Ltd (Tokyo, Japan). CCl_4_ was diluted to 10% with corn oil. Rifaximin was suspended in 0.5% Tween 80 and prepared to 10 mg/mL using a mortar and pestle.

### Experimental designs

A total of 35 eight-week-old mice were injected intraperitoneally with 2 μL/g bodyweight of diluted CCl_4_ dissolved in corn oil or the same volume of corn oil twice a week for 12 weeks. Thereafter, CCl_4_-treated mice received rifaximin (100 mg/kg/day) dissolved in Tween 80 or the same volume of Tween 80 for two weeks [19]. Corn oil-treated mice received only Tween 80. We evaluated liver fibrosis by Masson's trichrome stain.

### DNA extraction from intestinal components and stools

The duodenum was defined as 3 cm from the pylorus of the stomach. The remaining small intestine was further divided into halves, with the oral side defined as the jejunum and the aboral side as the ileum [22]. We squeezed out the intestinal contents with sterile tweezers and extracted the DNA.

DNA extraction was performed using the DNeasy PowerSoil Kit or its new version, the DNeasy PowerSoil Pro Kit (Qiagen, Hilden, Germany). Four mice did not defecate at the time of sacrifice and could not be analyzed (control group, n = 2; CCl_4_ group, n = 2; rifaximin group, n = 0).

### Construction of DNA Library and sequencing

The V3 and V4 regions of the 16S rRNA gene were amplified by nested PCR. First PCR was performed using KOD Fx Neo (TOYOBO, Osaka, Japan) at 94 °C for 2 min followed by 20 cycles at 98 °C for 10 s, 63 °C for 30 s, and 68 °C for 60 s. The forward primer was 5′- TCC TAC GGG NGG CWG CAG -3′, and the reverse primer was 5′- GTG GAC TAC HVG GGT ATC TAA TCC -3′. Using 1 μL of product from the first PCR as the template, a second PCR was performed using the KAPA Hifi HotStart ReadyMix (Kapa Biosystems, Wilmington, MA, USA) using 5′-ACA CGA CGC TCT TCC GAT CTC CTA CGG GNG GCW GCA G-3′ and 5′-GAC GTG TGC TCT TCC GAT CTG ACT ACH VGG GTA TCT AAT CC-3′ at 95 °C for 3 min followed by 20 cycles at 98 °C for 20 s, 50 °C for 30 s, and 72 °C for 60 s. We purified the PCR products using Agencourt AMPure XP magnetic beads (Beckman Coulter, Brea, CA, USA). Since the stool samples had a low concentration of bile acids and a large number of bacteria, only the second PCR was performed.

For library construction, PCR was performed with the KAPA Hifi HotStart ReadyMix using NEB Next Multiplex Oligos for Illumina (Dual Index Primers Set 1; NEB, Ipswich, MA, United States) at 95 °C for 3 min, and 8 cycles at 98 °C for 20 s, 50 °C for 30 s, and 72 °C for 60 s. The PCR products were purified using the Agencourt AMPure XP magnetic beads. Finally, a paired-end 2 × 300-bp cycle run was performed on an Illumina Miseq sequencing system (Illumina, San Diego, CA, USA).

### Sequence data analyses

We used Quantitative Insights Into Microbial Ecology version 2 (QIIME2) version 2020.6 [47] for sequencing analysis. We used DADA2 for merging and denoising paired-end reads [48]. Bacterial taxa were identified by comparing with the Greengenes database 13_8. Sequence reads attributed to the contaminated host’s DNA were removed by filter using Greengenes with 60% coverage and 65% identity similarity. The alpha diversity was estimated by the Shannon index, and the beta diversity was estimated by weighted Unifrac distances. Principal coordinate analysis was performed using STAMP ver 2.0 [49].

### Quantitative 16S rRNA PCR

The bacterial amount in the intestinal tract was evaluated by the quantitative PCR of the 16S rRNA gene. Premix Ex TaqTM (Probe qPCR; Takara, Shiga, Japan) and CSF96 real-time PCR analysis system (BIO-RAD, Hercules, CA, USA) were used. As a standard for the number of bacteria, we extracted DNA using the DNeasy PowerSoil Pro Kit from a solution of *Escherichia coli* for which colony-forming units had previously been measured. The probe was FAM-5' CGT ATT ACC GCG GCT GCT GCT GGC AC 3'-BHQ. The primer set was similar to that used for the first PCR of the 16S rRNA sequencing. The PCR reaction condition was 95 °C for 30 s followed by 40 cycles of 95 °C for 5 s and 63 °C for 30 s. The amount of DNA was normalized by the weight of the intestinal contents.

### Gut permeability test

Mice were fasted for 4 h, and 20 mL/kg of 4 kDa fluorescein isothiocyanate (FITC)-labeled dextran (Chondrex, Woodinville, WA, USA) was administered orally. Three hours later, mice were sacrificed and blood was collected from the left ventricle (control, n = 9; CCl_4_, n = 9; rifaximin, n = 10). The fluorescence intensity of the plasma was measured using a GloMax Multi Detection System (Promega, Madison, WI, USA) at an excitation wavelength of 490 nm/fluorescence wavelength of 520 nm.

### Statistical analysis

All statistical analyses were performed by comparing the control and CCl_4_ group to evaluate the effect of liver fibrosis and by comparing the CCl_4_ and rifaximin group to evaluate the effect of rifaximin.

We used the LEfSe method to compare the relative abundance of bacteria [50]. The threshold for LEfSe analysis was defined as a log-linear discriminant analysis (LDA) score > 3.5. The Mann-Whitney U test was used for most continuous variables. FITC dextran was normally distributed and was analyzed by Student’s t-test. Correlation analysis was performed by Spearman's rank correlation coefficient test. Statistical significance was defined as p values < 0.05. Statistical analysis was performed using R version 4.0.2.

## Supplementary Information

**Additional file 1: Figure S1.** Alpha rarefaction curve of each intestinal site. OTU, operational taxonomic units.

**Additional file 2: Table S1.** Bacteria with significant difference between the control group and CCl_4_ group in the duodenum. **Table S2.** Bacteria with significant difference between the CCl_4_ group and Rifaximin group in the duodenum. **Table S3.** Bacteria with significant difference between control group and CCl_4_ group in the jejunum. **Table S4.** Bacteria with significant difference between the CCl_4_ group and Rifaximin group in the jejunum. **Table S5.** Bacteria with significant difference between control group and CCl_4_ group in the ileum. **Table S6.** Bacteria with significant difference between the CCl_4_ group and Rifaximin group in the ileum. **Table S7.** Bacteria with significant difference between control group and CCl_4_ group in the cecum. **Table S8.** Bacteria with significant difference between the CCl_4_ group and Rifaximin group in the cecum. **Table S9.** Bacteria with significant difference between control group and CCl_4_ group in the stool. **Table S10.** Bacteria with significant difference between the CCl_4_ group and Rifaximin group in the stool.

## Data Availability

The read data have been deposited in GenBank under accession number DRA015190.

## Abbreviations

CCl_4_: Carbon tetrachloride
ETEC: Enterotoxigenic *Escherichia* *coli*
FITC: Fluorescein isothiocyanate
IBS: Irritable bowel syndrome
IQR: Interquartile rage
LEfSe: Linear discriminant analysis effect size
LDA: Linear discriminant analysis
NAFLD: Nonalcoholic fatty liver disease
OTU: Operational taxonomic unit
QIIME2: Quantitative Insights Into Microbial Ecology version 2
SIBO: Small intestinal bacterial growth
16S rRNA: 16S ribosomal RNA
