# Supplementary figures and images for: Modulation of duodenal and jejunal microbiota by rifaximin in mice with CCl4-induced liver fibrosis
Source: Gut Pathog. 2023 Mar 21;15:14. doi: 10.1186/s13099-023-00541-4 (PMC10029291; doi:10.1186/s13099-023-00541-4)

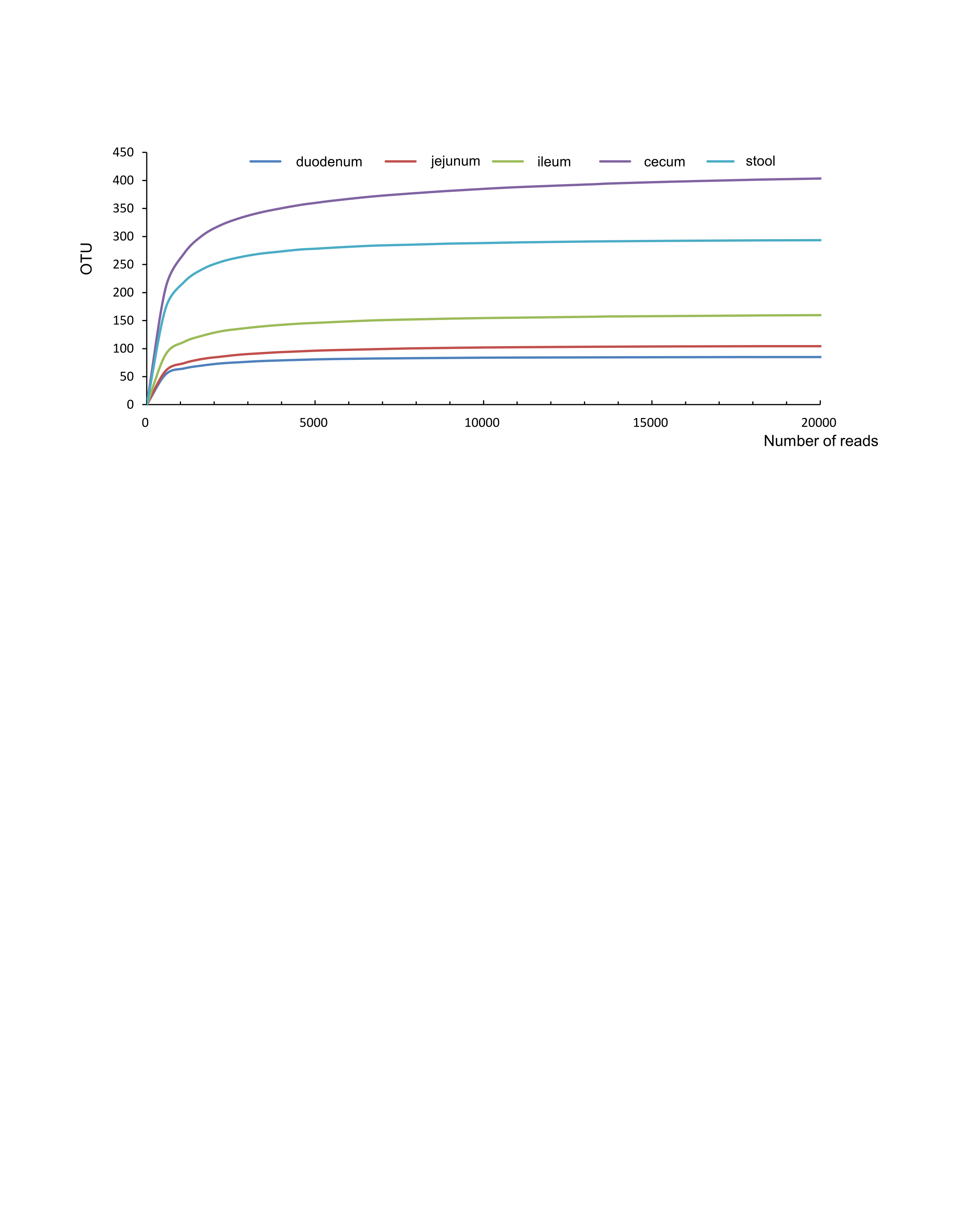

Supplement: Supplementary file 1 — Additional file 1: Figure S1. Alpha rarefaction curve of each intestinal site. OTU, operational taxonomic units. [file 13099_2023_541_MOESM1_ESM.tif]
