# Supplementary material for: Modulation of duodenal and jejunal microbiota by rifaximin in mice with CCl4-induced liver fibrosis
Source: Gut Pathog. 2023 Mar 21;15:14. doi: 10.1186/s13099-023-00541-4 (PMC10029291; doi:10.1186/s13099-023-00541-4)
Supplement: Supplementary file 2 — Additional file 2: Table S1. Bacteria with significant difference between the control group and CCl4 group in the duodenum. Table S2. Bacteria with significant difference between the CCl4 group and Rifaximin group in the duodenum. Table S3. Bacteria with significant difference between control group and CCl4 group in the jejunum. Table S4. Bacteria with significant difference between the CCl4 group and Rifaximin group in the jejunum. Table S5. Bacteria with significant difference between control group and CCl4 group in the ileum. Table S6. Bacteria with significant difference between the CCl4 group and Rifaximin group in the ileum. Table S7. Bacteria with significant difference between control group and CCl4 group in the cecum. Table S8. Bacteria with significant difference between the CCl4 group and Rifaximin group in the cecum. Table S9. Bacteria with significant difference between control group and CCl4 group in the stool. Table S10. Bacteria with significant difference between the CCl4 group and Rifaximin group in the stool. [file 13099_2023_541_MOESM2_ESM.doc]

Additional file 2: Table S1. Bacteria with significant difference between the control group and CCl_4_ group in the duodenum

| phylum | class | order | family | genus | species | Control group | CCl_4_ group | p value | LDA score |
| --- | --- | --- | --- | --- | --- | --- | --- | --- | --- |
| Firmicutes | Bacilli | Bacillales | Staphylococcaceae | *Staphylococcus* | - | 0% [0–0.009%] | 0% [0–0%] | 0.03 | 3.9 |
|  |  |  |  | - | - |  |  |  |  |
|  |  |  | - | - | - | 0.009% [0–0.2%] | 0% [0–0%] | 0.003 | 3.9 |
| The results of linear discriminant analysis effect size (LEfSe) method are shown. P value was estimated by the Mann-Whitney U test.  Bacteria with a log-linear discriminant analysis (LDA) score >3.5 and p value <0.5 are shown in the table.  Abbreviations: LDA, log-linear discriminant analysis | | | | | | | | | |

Additional file 2: Table S2. Bacteria with significant difference between the CCl_4_ group and Rifaximin group in the duodenum

| phylum | class | order | family | genus | species | CCl_4_group | Rifaximin group | p value | LDA score |
| --- | --- | --- | --- | --- | --- | --- | --- | --- | --- |
| Firmicutes | Bacilli | Lactobacillales | Lactobacillaceae | *Lactobacillus* | *reuteri* | 19.2% [9.4–22.7%] | 0% [0–4.6%] | 0.006 | 5.2 |
|  |  |  |  |  | unassigned *Lactobacillus* | 58.9% [43.1–65.0%] | 16.7% [14.4–46.4%] | 0.04 | 5.2 |
|  |  |  |  |  | - | 79.4% [52.9–90.8%] | 19.0% [15.4–75.4%] | 0.006 | 5.3 |
|  |  |  |  | - | - |  |  |  |  |
|  | - | - | - | - | - | 91.8% [79.7–94.2%] | 61.7% [30.9–86.2%] | 0.04 | 5.2 |
| The results of linear discriminant analysis effect size (LEfSe) method are shown. P value was estimated by the Mann-Whitney U test.  Bacteria with a log-linear discriminant analysis (LDA) score >3.5 and p value <0.5 are shown in the table.  Abbreviations: LDA, log-linear discriminant analysis | | | | | | | | | |

Additional file 2: Table S3. Bacteria with significant difference between control group and CCl_4_ group in the jejunum

| phylum | class | order | family | genus | species | Control group | CCl_4_ group | p value | LDA score |
| --- | --- | --- | --- | --- | --- | --- | --- | --- | --- |
| Firmicutes | Bacilli | Lactobacillales | Lactobacillaceae | *Lactobacillus* | unassigned *Lactobacillus* | 60.8% [27.1–69.4%] | 74.1% [59.4–79.5%] | 0.03 | 4.9 |
|  |  |  |  |  | - | 67.0% [32.2–78.6%] | 87.8% [67.5–90.6%] | 0.03 | 4.9 |
|  |  |  |  | - | - |  |  |  |  |
| The results of linear discriminant analysis effect size (LEfSe) method are shown. P value was estimated by the Mann-Whitney U test.  Bacteria with a log-linear discriminant analysis (LDA) score >3.5 and p value <0.5 are shown in the table.  Abbreviations: LDA, log-linear discriminant analysis | | | | | | | | | |

Additional file 2: Table S4. Bacteria with significant difference between the CCl_4_ group and Rifaximin group in the jejunum

| phylum | class | order | family | genus | species | CCl_4_ group | Rifaximin group | p value | LDA score |
| --- | --- | --- | --- | --- | --- | --- | --- | --- | --- |
| Firmicutes | Bacilli | Gemellales | Gemellaceae | *Gemella* | unassigned *Gemella* | 0% [0–0.3%] | 0% [0–0%] | 0.04 | 3.9 |
|  |  |  |  |  | - |  |  |  |  |
|  |  |  |  | - | - |  |  |  |  |
|  |  |  | - | - | - |  |  |  |  |
|  |  | Lactobacillales | Lactobacillaceae | *Lactobacillus* | *reuteri* | 8.0% [5.7–10.2%] | 0.7% [0–6.2%] | 0.009 | 4.4 |
|  |  |  |  |  | - | 87.8% [67.5–90.6%] | 61.3% [33.7–77.4%] | 0.03 | 5.1 |
|  |  |  |  | - | - |  |  |  |  |
| The results of linear discriminant analysis effect size (LEfSe) method are shown. P value was estimated by the Mann-Whitney U test.  Bacteria with a log-linear discriminant analysis (LDA) score >3.5 and p value <0.5 are shown in the table.  Abbreviations: LDA, log-linear discriminant analysis | | | | | | | | | |

Additional file 2: Table S5. Bacteria with significant difference between control group and CCl_4_ group in the ileum

| phylum | class | order | family | genus | species | Control group | CCl_4_ group | p value | LDA score |
| --- | --- | --- | --- | --- | --- | --- | --- | --- | --- |
| Firmicutes | Clostridia | Clostridiales | unassigned Clostridiales | - | - | 1.7% [1.3–1.9%] | 0.3% [0–0.8%] | 0.02 | 3.7 |
| The results of linear discriminant analysis effect size (LEfSe) method are shown. P value was estimated by the Mann-Whitney U test.  Bacteria with a log-linear discriminant analysis (LDA) score >3.5 and p value <0.5 are shown in the table.  Abbreviations: LDA, log-linear discriminant analysis | | | | | | | | | |

Additional file 2: Table S6. Bacteria with significant difference between the CCl_4_ group and Rifaximin group in the ileum

| phylum | class | order | family | genus | species | CCl_4_ group | Rifaximin group | p value | LDA score |
| --- | --- | --- | --- | --- | --- | --- | --- | --- | --- |
| Firmicutes | Clostridia | Clostridiales | Clostridiaceae | *CandidatusArthromitus* | unassigned *Candidatus Arthromitus* | 0.03% [0–3.5%] | 0% [0–0%] | 0.008 | 4.9 |
|  |  |  |  |  | - |  |  |  |  |
|  |  |  |  | - | - |  |  |  |  |
| TM7 | TM7_3 | CW040 | F16 | unassigned F16 | - | 1.4% [0.1–4.3%] | 0% [0–0.8%] | 0.05 | 4.0 |
|  | TM7_3 | CW040 | F16 | - | - |  |  |  |  |
|  | TM7_3 | CW040 | - | - | - |  |  |  |  |
|  | TM7_3 | - | - | - | - |  |  |  |  |
|  | - | - | - | - | - |  |  |  |  |
| The results of linear discriminant analysis effect size (LEfSe) method are shown. P value was estimated by the Mann-Whitney U test.  Bacteria with a log-linear discriminant analysis (LDA) score >3.5 and p value <0.5 are shown in the table.  Abbreviations: LDA, log-linear discriminant analysis | | | | | | | | | |

Additional file 2: Table S7. Bacteria with significant difference between control group and CCl_4_ group in the cecum

| phylum | class | order | family | genus | species | Control group | CCl_4_ group | p value | LDA score |
| --- | --- | --- | --- | --- | --- | --- | --- | --- | --- |
| Bacteroidetes | Bacteroidia | Bacteroidales | Bacteroidaceae | *Bacteroides* | *acidifaciens* | 2.1% [1.4–2.2%] | 2.7% [1.9–4.2%] | 0.04 | 3.7 |
|  |  |  |  | unassigned Rikenellaceae | - | 6.0% [3.5–6.8%] | 7.5% [6.7–9.2%] | 0.03 | 4.1 |
|  |  |  |  | - | - | 6.5% [3.7–7.4%] | 8.6% [7.2–9.6%] | 0.03 | 4.2 |
|  |  |  | - | - | - | 33.8% [30.3–35.6%] | 35.8% [35.3–41.9%] | 0.04 | 4.3 |
|  |  | - | - | - | - |  |  |  |  |
|  | - | - | - | - | - |  |  |  |  |
| Deferribacteres | Deferribacteres | Deferribacterales | Deferribacteraceae | *Mucispirillum* | *schaedleri* | 11.4% [7.4–17.5%] | 5.0% [0–10.2%] | 0.04 | 4.5 |
|  |  |  |  |  | - |  |  |  |  |
|  |  |  |  | - | - |  |  |  |  |
|  |  |  | - | - | - |  |  |  |  |
|  |  | - | - | - | - |  |  |  |  |
|  | - | - | - | - | - |  |  |  |  |
| Proteobacteria | Deltaproteobacteria | Desulfovibrionales | Desulfovibrionaceae | *Desulfovibrio* | - | 0.6% [0.5–0.9%] | 1.3% [0.9–2.0%] | 0.02 | 3.7 |
| The results of linear discriminant analysis effect size (LEfSe) method are shown. P value was estimated by the Mann-Whitney U test.  Bacteria with a log-linear discriminant analysis (LDA) score >3.5 and p value <0.5 are shown in the table.  Abbreviations: LDA, log-linear discriminant analysis | | | | | | | | | |

Additional file 2: Table S8. Bacteria with significant difference between the CCl_4_ group and Rifaximin group in the cecum

| phylum | class | order | family | genus | species | CCl_4_ group | Rifaximin group | p value | LDA score |
| --- | --- | --- | --- | --- | --- | --- | --- | --- | --- |
| Bacteroidetes | Bacteroidia | Bacteroidales | Bacteroidaceae | *Bacteroides* | *acidifaciens* | 2.7% [1.9–4.2%] | 6.1% [4.3–8.6%] | 0.04 | 4.2 |
|  |  |  |  |  | - | 5.3% [4.5–5.7%] | 9.8% [6.4–11.4%] | 0.02 | 4.3 |
|  |  |  |  | - | - |  |  |  |  |
|  |  |  | Rikenellaceae | unassigned Rikenellaceae | - | 7.5% [6.7–9.2%] | 5.5% [4.2–6.2%] | 0.003 | 4.1 |
|  |  |  |  | - | - | 8.6% [7.2–9.6%] | 5.5% [4.4–6.3%] | 0.002 | 4.2 |
| Firmicutes | Clostridia | Clostridiales | Ruminococcaceae | *Oscillospira* | unassigned *Oscillospira* | 5.1% [2.6–6.6%] | 9.2% [8.1–10.2%] | 0.0003 | 4.4 |
|  |  |  |  |  | - |  |  |  |  |
|  |  |  |  | - | - | 9.4% [5.8–11.8%] | 12.6% [11.0–14.5%] | 0.03 | 4.3 |
| TM7 | TM7_3 | CW040 | F16 | unassigned F16 | - | 1.3% [0.8–2.2%] | 0.3% [0.02–0.9%] | 0.009 | 3.6 |
|  |  |  |  | - | - |  |  |  |  |
|  |  |  | - | - | - |  |  |  |  |
|  |  | - | - | - | - |  |  |  |  |
|  | - | - | - | - | - |  |  |  |  |
| The results of linear discriminant analysis effect size (LEfSe) method are shown. P value was estimated by the Mann-Whitney U test.  Bacteria with a log-linear discriminant analysis (LDA) score >3.5 and p value <0.5 are shown in the table.  Abbreviations: LDA, log-linear discriminant analysis | | | | | | | | | |

Additional file 2: Table S9. Bacteria with significant difference between control group and CCl_4_ group in the stool

| phylum | Class | order | family | genus | species | Control group | CCl_4_ group | p value | LDA score |
| --- | --- | --- | --- | --- | --- | --- | --- | --- | --- |
| Bacteroidetes | Bacteroidia | Bacteroidales | Paraprevotellaceae | *Prevotella* | unassigned *Prevotella* | 2.5% [1.8–2.8%] | 0.8% [0.6–1.6%] | 0.008 | 3.9 |
|  |  |  |  |  | - |  |  |  |  |
|  |  |  |  | - | - | 2.5% [1.8–2.8%] | 0.8% [0.6–1.7%] | 0.01 | 3.9 |
|  |  |  | Bacteroidaceae | *Bacteroides* | *acidifaciens* | 4.4% [3.7–6.2%] | 9.7% [7.9–13.6%] | 0.0004 | 4.5 |
|  |  |  |  |  | - | 8.4% [6.8–11.0%] | 13.7% [10.9–18.6%] | 0.02 | 4.5 |
|  |  |  |  | - | - |  |  |  |  |
|  |  |  | Rikenellaceae | unassigned Rikenellaceae | - | 6.1% [4.6–7.3%] | 13.5% [9.5–19.1%] | 0.002 | 4.6 |
|  |  |  |  | - | - | 6.5% [5.0–7.8%] | 14.4% [10.2–20.0%] | 0.002 | 4.7 |
|  |  |  | S24_7 | unassigned S24_7 | - | 28.4% [24.1–29.8%] | 20.5% [13.9–24.3%] | 0.03 | 4.6 |
|  |  |  |  | - | - |  |  |  |  |
| Firmicutes | Bacilli | Bacillales | - | - | - | 0% [0–0.02%] | 0% [0–0%] | 0.03 | 3.6 |
|  |  | Lactobacillales | Streptococcaceae | *Streptococcus* | unassigned *Streptococcus* | 0% [0–0.04%] | 0% [0–0%] | 0.03 | 3.7 |
|  |  |  |  |  | - |  |  |  |  |
| Proteobacteria | - | - | - | - | - | 6.2% [5.1–7.5%] | 3.3% [2.3–5.3%] | 0.04 | 4.0 |
| The results of linear discriminant analysis effect size (LEfSe) method are shown. P value was estimated by the Mann-Whitney U test.  Bacteria with a log-linear discriminant analysis (LDA) score >3.5 and p value <0.5 are shown in the table.  Abbreviations: LDA, log-linear discriminant analysis | | | | | | | | | |

Additional file 2: Table S10. Bacteria with significant difference between the CCl_4_ group and Rifaximin group in the stool

| phylum | class | order | family | genus | species | CCl_4_ group | Rifaximin group | p value | LDA score |
| --- | --- | --- | --- | --- | --- | --- | --- | --- | --- |
| Bacteroidetes | Bacteroidia | Bacteroidales | Rikenellaceae | - | - | 14.4% [10.2–20.0%] | 9.3% [6.3–13.4%] | 0.04 | 4.5 |
| Deferribacteres | Deferribacteres | Deferribacterales | Deferribacteraceae | *Mucispirillum* | *schaedleri* | 0.3% [0–0.4%] | 0.8% [0.3–1.4%] | 0.05 | 3.8 |
|  |  |  |  |  | - |  |  |  |  |
|  |  |  |  | - | - |  |  |  |  |
|  |  |  | - | - | - |  |  |  |  |
|  |  | - | - | - | - |  |  |  |  |
|  | - | - | - | - | - |  |  |  |  |
| Firmicutes | Clostridia | Clostridiales | Ruminococcaceae | *Oscillospira* | unassigned *Oscillospira* | 5.1% [3.1–7.4%] | 10.6% [8.2–12.1%] | 0.004 | 4.4 |
|  |  |  |  |  | - |  |  |  |  |
|  |  |  | unassigned Clostridiales | - | - | 7.9% [5.3–10.4%] | 5.0% [3.6–5.9%] | 0.02 | 4.1 |
| TM7 | TM7_3 | CW040 | F16 | unassigned F16 | - | 4.0% [2.5–6.4%] | 0.7% [0.2–2.6%] | 0.007 | 4.2 |
|  |  |  |  | - | - |  |  |  |  |
|  |  |  | - | - | - |  |  |  |  |
|  |  | - | - | - | - |  |  |  |  |
|  | - | - | - | - | - |  |  |  |  |
| The results of linear discriminant analysis effect size (LEfSe) method are shown. P value was estimated by the Mann-Whitney U test.  Bacteria with a log-linear discriminant analysis (LDA) score >3.5 and p value <0.5 are shown in the table.  Abbreviations: LDA, log-linear discriminant analysis | | | | | | | | | |
